# Predicting Mechanical Thrombectomy Outcome and Time Limit through ADC Value Analysis: A Comprehensive Clinical and Simulation Study Using Machine Learning

**DOI:** 10.3390/diagnostics13132138

**Published:** 2023-06-21

**Authors:** Daisuke Oura, Soichiro Takamiya, Riku Ihara, Yoshimasa Niiya, Hiroyuki Sugimori

**Affiliations:** 1 Department of Radiology, Otaru General Hospital, Otaru 047-0152, Japan; 2 Graduate School of Health Sciences, Hokkaido University, Sapporo 060-0812, Japan; 3 Department of Neurosurgery, Otaru General Hospital, Otaru 047-0152, Japan; 4 Faculty of Health Sciences, Hokkaido University, Sapporo 060-0812, Japan

**Keywords:** acute ischemic stroke, MRI, ADC, machine learning

## Abstract

Predicting outcomes after mechanical thrombectomy (MT) remains challenging for patients with acute ischemic stroke (AIS). This study aimed to explore the usefulness of machine learning (ML) methods using detailed apparent diffusion coefficient (ADC) analysis to predict patient outcomes and simulate the time limit for MT in AIS. A total of 75 consecutive patients with AIS with complete reperfusion in MT were included; 20% were separated to test data. The threshold ranged from 620 × 10^−6^ mm^2^/s to 480 × 10^−6^ mm^2^/s with a 20 × 10^−6^ mm^2^/s step. The mean, standard deviation, and pixel number of the region of interest were obtained according to the threshold. Simulation data were created by mean measurement value of patients with a modified Rankin score of 3–4. The time limit was simulated from the cross point of the prediction score according to the time to perform reperfusion from imaging. The extra tree classifier accurately predicted the outcome (AUC: 0.833. Accuracy: 0.933). In simulation data, the prediction score to obtain a good outcome decreased according to increasing time to reperfusion, and the time limit was longer among younger patients. ML methods using detailed ADC analysis accurately predicted patient outcomes in AIS and simulated tolerance time for MT.

## 1. Introduction

Cerebral vascular diseases are commonly confronted in the neuro-emergency field. Stroke is the most common serious manifestation of cerebrovascular disease. Stroke is the fifth-leading cause of death in the fourth in Japan, and a major cause of severe disability. According to the 2020 American Heart Association’s report on Heart Disease and Stroke Statistics, it was estimated that approximately 2.5% of the population in the United States experienced a stroke in 2016. This equates to around 7 million Americans aged 20 years or older who had suffered from a stroke, resulting in nearly 800,000 stroke incidents and approximately 150,000 deaths. Age is the primary demographic factor that contributes to the risk of stroke, and although the incidence of stroke has decreased in recent years, the lifetime risk of stroke has risen due to the aging population. Additional risk factors include being female and belonging to the African American race. The estimated cost of stroke for the year 2014–2015 was $45.5 billion [1].

Cerebral infarction is the main characteristic of ischemic stroke. When there is insufficient blood supply to the cerebral tissue, the first stage involves a reversible decline in tissue function. If this condition persists, it progresses to infarction, resulting in the loss of neurons and supportive structures. Ischemia triggers a series of events, starting with the loss of electrical function and leading to membrane dysfunction. This dysfunction involves an influx of calcium, which induces calcium-dependent excitotoxicity, the production of reactive oxygen species, and ultimately the breakdown of cell membranes and cell lysis [2,3,4].

Cerebral infarction has several different mechanisms [5]. Cardio embolism, which involves the formation of blood clots in the heart, is the prevailing cause of stroke. Most emboli originate from the heart and are typically associated with cardiac conditions. Examples of common heart disorders that increase the risk of stroke include atrial fibrillation, valvular heart disease, and cardiomyopathy resulting from myocardial infarction or hypertension. Although less frequent, other factors can also contribute to cardiomyopathy. In acute cardioembolism, the main cerebral arteries are often occluded by a blood clot. Another prevalent underlying cause of stroke is large vessel disease. This type of disease is commonly associated with atherosclerosis in the proximal cervical internal carotid arteries, although it can also occur in more distal parts of the internal carotid arteries, the aorta, the vertebral and basilar arteries, or intracranially. The second most common cause of large vessel disease is arterial dissection, which typically affects the internal carotid or vertebral arteries. Arterial dissection is often responsible for strokes in young patients who do not have other risk factors, as well as in individuals with specific predisposing conditions. These acute large vessel occlusions are called Acute ischemic stroke (AIS).

The development of irreversible infarction in cerebral tissue is influenced by the degree and duration of the decline in cerebral blood flow. When cerebral blood flow drops by approximately 50%, patients generally do not experience any symptoms. However, as the flow further decreases, reversible dysfunction of neurons takes place, resulting in ischemic symptoms that typically manifest as functional deficits corresponding to the affected area. If blood flow is restored promptly enough, neuronal function can return without any infarction, which is referred to as a transient ischemic attack [2,6,7]. However, if low blood flow leading to ischemia persists for a significant duration, irreversible tissue damage occurs, triggering the same pathophysiological processes observed in cerebral infarction or ischemic stroke. The time it takes for irreversible tissue injury to develop from the onset of symptoms is dependent on the extent and duration of the decline in cerebral blood flow. The acute management of stroke focuses on the timely reperfusion of at-risk tissue through intravenous thrombolysis or endovascular thrombectomy, as well as the optimization of hemodynamic status by carefully managing fluid volume, blood pressure, and cardiovascular health. A significant breakthrough in acute stroke care was the use of intravenous tissue plasminogen activator (tPA, alteplase) for acute ischemic stroke. While IV tPA is generally beneficial, it may not effectively treat many patients, particularly those with occlusions in proximal large vessels such as the middle cerebral artery (MCA) or internal carotid artery (ICA) [8,9].

Although traditional treatment methods such as thrombolytics have been used to restore blood flow in AIS, mechanical thrombectomy (MT) has emerged as a highly effective alternative in recent years [10,11,12,13,14,15]. Time to recanalization is crucial in MT for AIS, with the best results achieved when the procedure is performed as early as possible after symptom onset [16]. Patients who exhibit substantial functional deficits, along with a large vessel occlusion, and present within 6 h of symptom onset, without evidence of a significant stroke on CT or MRI scans, and without contraindications, should be evaluated for endovascular thrombectomy. Additionally, certain selected patients who meet specific criteria, even beyond the initial 6-h window, may also be considered for this intervention, with the timeframe extended up to 24 h. The decision to proceed with late-window (>6 h) intervention is based on imaging assessments that confirm the presence of salvageable tissue at risk and a relatively small established core infarct. It is through these imaging findings that patients can be identified and selected for the appropriate course of treatment. MT involves minimally invasive procedures to remove blood clots, particularly in cases of large-vessel occlusions, resulting in improved outcomes, and functional recovery.

Efforts to streamline the process of stroke care and reduce treatment times for MT have led to advancements in procedural techniques and technologies, such as stent retrievers and improved imaging analysis [17,18]. Despite these efforts, achieving favorable clinical outcomes in AIS, such as a modified Rankin score (mRS) of 0–2, has remained challenging. Thus, knowledge of the time limit from imaging to obtain a good outcome will greatly assist in the decision-making process of performing MT at the time of imaging examination and carries great clinical merit. In particular, the time boundaries between poor and good outcomes will be a crucial factor in clinicians’ decisions to apply MT.

To address this challenge, we focused on the apparent diffusion coefficient (ADC) value and machine learning (ML) as potential solutions. The ADC value is calculated from diffusion-weighted imaging (DWI) using two different b-values in magnetic resonance imaging (MRI). The ADC value is a readily accessible quantitative value for assessing brain tissue in the acute stage of AIS, even immediately after onset, and has been used to assess AIS. DWI and ADC sequences demonstrate an almost 100% sensitivity in the detection of acute infarction. The presence of bright lesions on DWI and dark lesions on ADC, without early changes on FLAIR, typically indicates acute strokes captured within approximately 6 h from the onset [19,20,21].

ML is a powerful tool in the artificial intelligence (AI) field for analyzing complex relationships among various factors and has been widely used in medical applications, including outcome prediction [22,23,24]. AI is useful in triaging, diagnosing, patient selection, and outcome prediction of large vessel occlusion (LVO), but its application is still in the exploratory stage. Most literature focuses on diagnosis and demonstrates reasonably high accuracy. The future priority of AI applications should shift beyond diagnosis to optimizing treatment through more nuanced patient selection for mechanical thrombectomy (MT) and predicting clinical and angiographic outcomes associated with it [22].

By leveraging ML techniques in obtaining ADC values, it may be possible to accurately classify patient outcomes after MT. Moreover, the change in prediction score according to the time process may allow us to simulate the time limit in which good outcomes can be obtained.

Such predictive models could optimize patient selection for MT, leading to improved clinical outcomes and better resource allocation in stroke care. Hence, this study aims to investigate whether ML methods using detailed ADC analysis can accurately predict patient outcomes in AIS and whether these models can simulate the tolerance time for MT.

## 2. Materials and Methods

This retrospective cohort study was carried out following the guidelines set forth in the Declaration of Helsinki and received approval from the Institutional Review Board (IRB) of Otaru General Hospital (IRB approval 04-019). Prior to their involvement in the study, all patients or their respective families provided informed consent either orally or in writing. The authors of this study declare that they have no conflicts of interest.

### 2.1. Patients

We included consecutive AIS patients with occlusion sites in the anterior circulation who underwent MRI as their first imaging examination and endovascular treatment immediately after arrival in our institution between January 2016 and December 2021.

To eliminate the impact of technical differences in the outcome, we targeted patients who achieved complete reperfusion with thrombolysis in cerebral infarction grade 3 (TICI 3). Patients who did not obtain complete reperfusion such as TICI 1 or 2 were excluded because these patients did not avoid an impact of the remaining infarcted region. Additionally, patients who had an intra-cerebral hemorrhage in pre-post image examination were excluded.

Detailed patient information was obtained from medical records. The following data were obtained; patient’s age, time to re-perfusion time from the imaging examination, number of passes to achieve TICI3, mRS in pre-AIS, National Institutes of Health Stroke Scale (NIHSS) score in pre-AIS, Alberta Stroke Program Early CT Score (ASPECTS). ASPECTS was manually identified using DWI with b = 1000 s/mm^2^ by the neurosurgeon. We defined the patient outcome as either the mRS 3 months after onset or at the time of discharge.

### 2.2. Image Acquisition

All MRIs were acquired using a 3-Tesla machine with a 32-channel head coil (Ingenia 3.0T, Philips Healthcare, Best, The Netherlands). The DWI parameters were as follows: sequence was single-shot spin-echo–echo-planar imaging; repetition time was in the range of 2000 milliseconds to 5000 msec; echo time was 79 msec; field of view was 230 mm; spatial resolution was 1.8 × 2.34 mm; Flip angle was 90 degree; slice thickness was 5 mm; slice number was 22; parallel imaging factor was 3; and scan time was 14 s. Other acquired sequences were the following; arterial spin labeling, head and neck magnetic resonance angiography, fluid-inversion recovery (FLAIR), T2* weighted imaging, and chest coronal MRA. The protocol for AIS was performed for a total of about 10 min of actual scan time.

### 2.3. ADC Analysis

**Figure 1:**
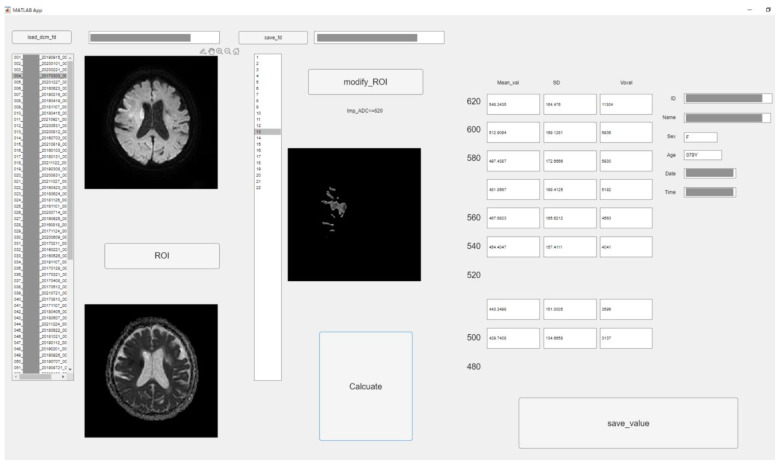
The graphic user interface of original software in MATLAB. According to threshold of ADC value (620 × 10^−6^ mm^2^/s–480 × 10^−6^ mm^2^/s, 20 × 10^−6^ mm^2^/s step), low ADC value area was semi-automatically segmented. The mean, standard deviation (SD), and voxel number were measured of each ROI. (Personal information was masked).

To perform the detailed evaluation of the ADC value, we developed original graphic user interface software using in-house MATLAB software (The Mathworks, Inc., Natick, MA, USA). Figure 1 depicts the software structure. This software allows semiautomatic analysis according to the threshold of ADC. The procedure was as follows: at first, analysts roughly drew circular regions of interest (ROIs) covering high signal areas in DWI and low value in ADC or occlusion sites. Then, ROIs with ADC values less than 620 × 10^−6^ mm^2^/s were created by automatically processing according to the threshold roughly ROI in the above. The extra parts were removed manually with care. All 22 slices were used for ADC analysis.

We could obtain all pixel values of each ROI corresponding to each threshold using MATLAB software. Therefore, the sum or average of them is simply obtained. Therefore, upon pressing the calculate button, the values corresponding to each ADC value were calculated from the ROI, and the mean, standard deviation (SD), and pixel number were measured with each threshold. 

The ADC threshold was used in increments of 20 × 10^−6^ mm^2^/s from 620 × 10^−6^ mm^2^/s to 480 × 10^−6^ mm^2^/s. All values were saved into a comma-separated value file. Two experienced radiographers with 21 and 9 years of experience performed the measurements under an individual separate setting. Intraclass correlation coefficients (ICCs) were calculated for all measurement values. 

Eight thresholds were used. Additionally, ROIs were created corresponding to each threshold. The fusion images were created in the following steps. Mask image which had 0 or 1 signal was created according to ROIs. Eight mask images were accumulated into one image. Therefore, each pixel of accumulated images would have a maximum value of 8 and a minimum value of 0. This pixel value was displayed in steps by color and fused with a b = 1000 image.

**Figure 2:**
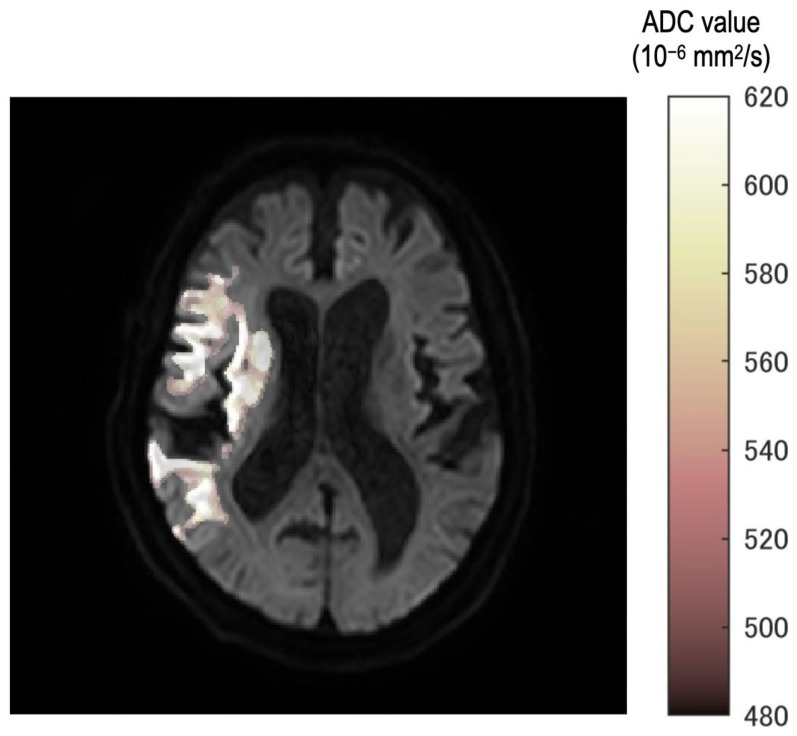
The fusion image ROI of each threshold and b = 1000 image. Stepwise color tone changes can be observed according to the ADC value. All images were reviewed by the neurosurgeon for any differences from the occlusion point.

In all cases, the neurosurgeon confirmed the consistency between the occlusion point and ROI using the fusion image (Figure 2). Furthermore, fusion images between DWI with b = 1000 s/mm^2^ and each ROI of the ADC value were created automatically. 

In the fusion image, the discrepancy of threshold was described as a gradual color change from white to deep red. Therefore, observers can understand the discrepancy as texture according to the threshold in the ischemic region.

### 2.4. Data Handling and ML

At first, we divided patients into two groups which were the good outcomes group and the poor outcome group. We defined an mRS of 0–2 as indicating a good outcome and any score greater than 3 as indicating a poor outcome. We attempted to construct a classification model to distinguish between good and poor outcomes using ML methods. We used Python 3.8.6 (Jupyter Lab and the Pycaret Library) for data handling and construction of ML models.

Initially, patients were randomly split into 80% training data and 20% testing data groups. We performed data augmentation on the training data by reversing the occlusion site from left to right and vice-versa. In addition, we performed synthetic data generated using the ctgan method. The sample size of the training data was increased to 220 cases. Normalization was performed using the robust method. To address the imbalance in data. we also applied the synthetic minority oversampling technique (SMOTE). The feature selection threshold was set to 0.80 [25]. The following features were used for the construction of the classification ML models: patients’ age, sex, occlusion location (right or left), time to reperfusion from imaging, mean of ADC, SD of ADC, and pixel number of region. In total, we used 28 features to create the ML models. All features were used as continuous values.

The ML algorithms were used to identify significant models. We compared the performance of 15 ML algorithms based on accuracy, the area under the curve (AUC), recall, precision, and F1 scores by validation data. To create a single model for predicting the output, a voting classifier ML model was also constructed by combining the two best models. We applied five-fold cross-validation in all model construction processes. The importance of features was confirmed in the top two models. The top three features were then compared between the good and poor outcomes.

We then evaluated the two best and the created blend models in terms of accuracy and AUC on the test set. The confusion matrix provided AUC, accuracy, recall, precision, and F1 scores.

### 2.5. Simulation to Estimate the Time Limit for MT

We conducted a simulation study to investigate the time limit to obtain good outcomes by the MT. The simulation data were created from the average values of overall patients with mRS 3 or 4 in the same cohort group of the model construction. The simulation data with three different ages were prepared to investigate the difference in outcomes due to age. Three ages were mean values and ±SD. The other parameters were set as follows: sex, male; occlusion site, right. The time to reperfusion from imaging was changed from 10 to 360 min. Prediction scores were calculated according to the time to reperfusion from the imaging, using the best model which was created in the above studies. The relationship between time to reperfusion from imaging and prediction score, the time limit was defined from the cross point of the prediction score in both good and poor outcomes. We evaluated the time limit from the relationship between time and prediction scores. The time limit for MT was defined as the cross point of the prediction score in both good and poor outcomes. Differences in time limit by age and trends in the dynamics of the prediction score were confirmed.

### 2.6. Statistical Analysis

We used the Wilcoxon signed test to compare the performance of the ML model and each value in the good outcome group and the poor outcome group. A *p*-value < 0.05 was considered statistically significant. We used R version 4.1.1 for all statistical analyses and figure creation.

## 3. Results

### 3.1. Participants and ADC Value Analysis

Table 1 presents the summary of participants; 75 patients met our criteria. The mean age was 79.9 years, and the time to reperfusion (im2P) was 121.6 min. The mean number of passes to obtain TICI 3 was 1.8.; The mean pre- and post-mRS were 0.8 and 3.5.; The mean NIHSS was 17.6.; The mean ASPECTS was 8.1. Further detailed patient information was shown in the Supplementary Materials (Table S1).

Table 2 shows the summary of the ADC value analysis. In all values, ICCs were higher than 0.95.

### 3.2. Comparison of the Performance of ML Models to Estimate Patient Outcomes

Table 3 presents a comparison of the performance of the ML models for validation. The best-performing model was the extra tree classifier, and the second was the random forest tree. Table 4 shows the performance of the best two models after parameter tuning for AUC.

**Figure 3:**
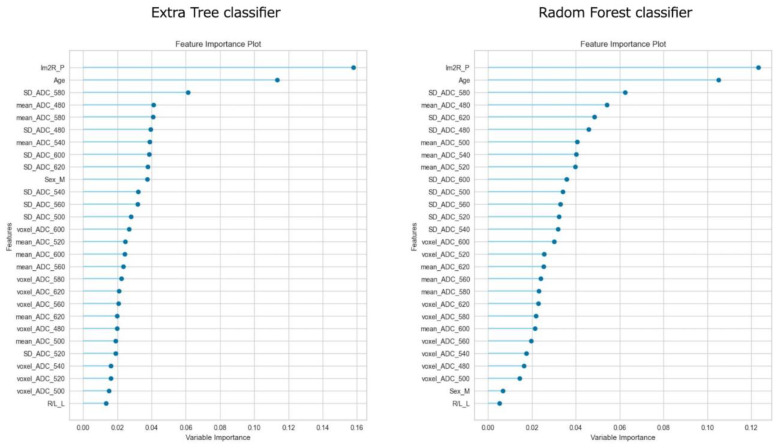
The feature importance of top two models.

**Figure 4:**
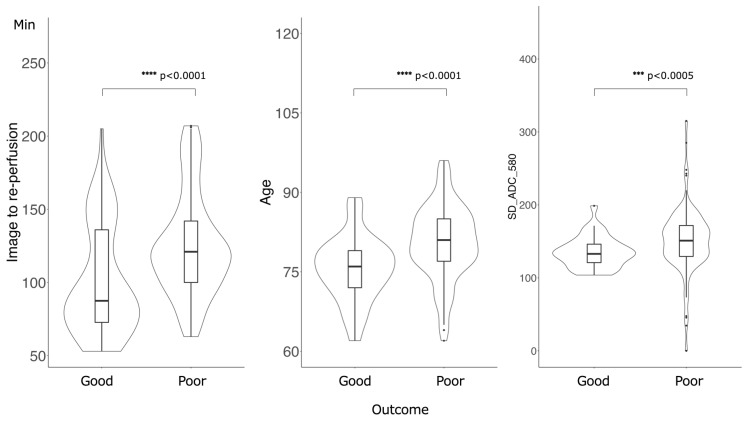
Comparison of the values of the important features between good outcome groups and poor outcome groups. There was a significant difference in all values: Time of image to re-perfusion, Age and SD of ADC ≤ 580 × 10^−6^ mm^2^/s.

**Figure 5:**
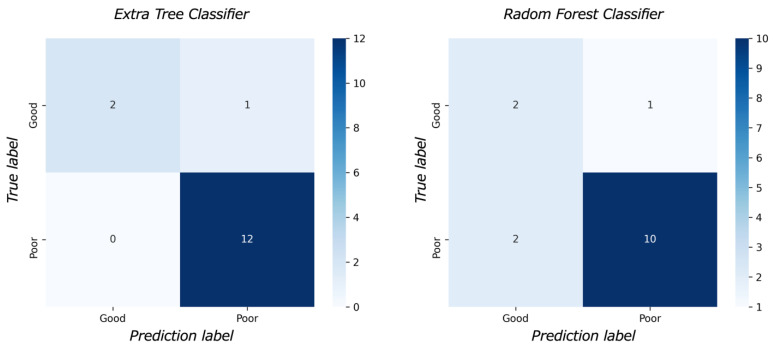
The confusion matrix.

Figure 3 shows the feature importance of each model. In both models, the most important feature was time to reperfusion, the second most important feature was patient age, and the third most important was SD of ADC ≤ 580 × 10^−6^ mm^2^/s. In comparison to each value between the good outcome group and the poor outcome group, there was a significant difference in all values between the good and poor outcome groups (Figure 4). Figure 5 presents the confusion matrix of the best two models on the test data. Furthermore, Table 5 displays the performance of each model for the test data sets. The extra tree classifier demonstrated the best performance (accuracy, 0.933; AUC, 0.833, Recall, 0.667, F1 score, 0.800).

### 3.3. Simulation to Estimate the Time Limit for MT

**Figure 6:**
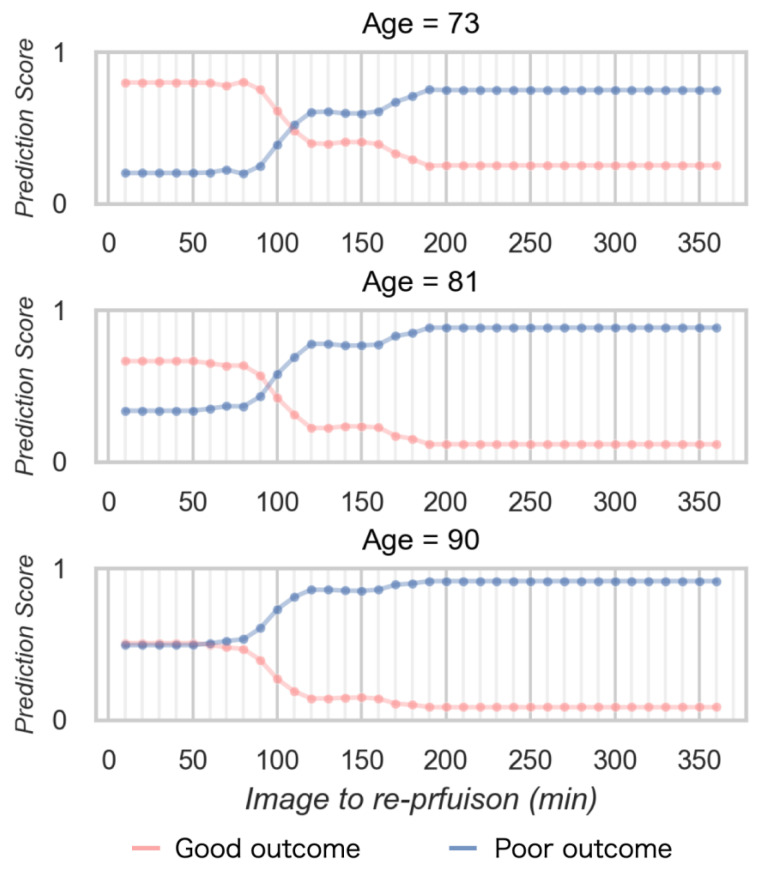
The relationship between the prediction score and time to reperfusion in the simulation data.

Figure 6 shows the relationship between the prediction score and time to reperfusion in the simulation data. The prediction score for obtaining a good outcome decreased according to increasing time to reperfusion from imaging. The time limit demonstrate as the cross point was longer for young age, and shorter for older age. The prediction score to obtain a good outcome was lower according to aging.

## 4. Discussion

This study introduced a novel approach that uses ML to estimate patient outcomes and a time limit for MT in AIS. ADC values are the most accessible quantitative value and worked to assess patient outcomes and the time limit to reperfusion by mechanical thrombectomy after imaging. Our results demonstrated that the ML model using the detailed ADC analysis achieved high accuracy and AUC in predicting outcomes.

Previous studies have demonstrated that time to reperfusion is a crucial factor in achieving better clinical outcomes, with worse outcomes observed as the time to reperfusion increases. In AIS patients due to LVO, 32,000 neurons are lost per second, 8.7 h of aging per second [26]. This dramatic change is directly related to patient outcomes and is the main reason why “time is brain” is used as a keyword in AIS treatment. ADC values might reflect such a drastic change in brain tissue after the onset of LVO. In the keyword “Time is brain”, accessibility is an important factor in clinical use, and it is considered that ADC values were suitable to construct ML models to evaluate the acute stage of AIS. 

Age also plays a role in AIS treatment outcomes, with older age groups experiencing more difficulty in achieving good outcomes. It is reported that a very poor three-month outcome was independently associated with higher admission NIHSS, absence of thrombolysis, absence of recanalization, and higher frailty status in elderly patients over 80 years old [27,28]. This study supports these findings, with both times to reperfusion and age identified as important predictors of outcomes. In this study, all patients obtained complete recanalization, the age of the poor outcome group is significantly higher, implying that obtaining a good prognosis remains difficult for the elderly.

In our cohort group, there was a significant difference between a good outcome group and a poor outcome group in time to re-perfusion from imaging and age. This result demonstrated the same trend as previous studies. Moreover, SD of ADC ≤580 × 10^−6^ mm^2^/s worked as a third important feature. This result means that ADC value will work as one of the predictive factors of patient outcome in AIS as mentioned in the previous studies.

In the simulation data, the prediction score of good outcomes decreased according to time elapsed and higher age and that of poor outcomes increased according to age. Younger age demonstrated a longer time limit to obtain good outcomes. Basically, these findings which were obtained using the ML model demonstrated a similar trend to previous studies. In other words, the ML model which was constructed from the detailed ADC analysis integrated previous studies and allowed to immediately predict individual patient outcomes.

Prediction of outcomes and the time limit for MT provide some clinical merits. Our results contribute to the decision as to whether to apply MT for each patient. For instance, 25% of all strokes occur during sleep [29]. So called, “Wake-up stroke”. In these patients, the accurate time from the onset cannot be known. In clinical practice, we often face difficulty to decide the treatment strategy for these patients. In other words, whether MT should be applied for wake-up ischemic stroke is a major concern. FLAIR-DWI mismatch provides important information to use rt-PA. Patients with DWI-FLAIR mismatch can be presumed to be within 3 h of onset and can therefore receive rt-PA [30]. However, the effects of rt-PA on the internal carotid artery and proximal main trunk occlusion are known to be limited, and it is difficult to say that serious disability can be avoided in AIS due to LVO. On the other hand, excessive medical care should be avoided in terms of cost and surgical risk, and this boundary is always a source of concern.

In this study, we used the time to reperfusion from MRI as one of the features, and it worded an important factor in estimating the outcome. This result may help resolve a serious problem related to the onset time, such as a wake-up stroke. Because the time between the imaging examination and MT is always clear. Additionally, when AIS occurs alone, the last healthy time is often unclear due to unconsciousness. The use of time from imaging examination resolves this problem and may be clinically useful. If the patient outcomes can be estimated immediately after the imaging examination, it may be possible to avoid MT for patients with low indications for treatment and reduce healthcare costs. Moreover, the prediction of the time limit for MT offers clear time goals until recanalization and allowed for avoiding unnecessary prolongation of MT. This also works to release a medical resource.

Previous investigations have identified the ADC value as an important factor in predicting a patient’s outcome. Perhaps ADC analysis accurately reflects the brain tissue condition on imaging examination. Purushotham et al. identified a threshold ADC value of <620 × 10^−6^ mm^2^/s for the ischemic core in AIS [31]. Gwak et al. investigated the region volume using several thresholds and reported that a low value of the ratio between <520 × 10^−6^ mm^2^/s and 620 × 10^−6^ mm^2^/s reflects less severe ischemic stress inside a diffusion lesion in a large ischemic core and may predict clinical outcome [32]. In addition, Yu et al. reported that ADC values worked as an individual prediction factor to predict the progression of perforator artery cerebral infarction. There was a significant difference in the mean ADC values of the ischemic region between the progress and nonprogress groups, but an overlap of ADC values was also confirmed in their study. Less than 620 × 10^−6^ mm^2^/s has been used as a standard to estimate the ischemic core volume in ADC analysis; however, it is known that this does not perfectly reflect the core region [33]. Umemura et al. reported that a value >520 × 10^−6^ mm^2^/s can be salvageable by MT, and the high signal in DWI is reversible [34]. Thus, ADC thresholds for predicting patient outcomes vary, and there is no golden standard. A definitive ADC value that can serve as a predictive factor for the outcome remains unknown.

From these aspects, we performed the detailed ADC analysis from 620 × 10^−6^ mm^2^/s to 480 × 10^−6^ mm^2^/s with semiautomatic measurements using the original MATLAB software, and attempted the ML method to reveal the complex relationship between ADC values and outcomes. As a result, in both of the best two models, an SD of <580 × 10^−6^ mm^2^/s was the third important feature. This result showed that ADC values also worked as an important feature to predict outcomes. Hence, combining the detailed ADC analysis and ML method may be a good way to cover the conventional ADC value measurement with manual measurement. It is possible to incorporate the ML model into the software for ADC measurement, which means that immediate analysis can estimate patient prognosis. The time to reperfusion from imaging was not considered in previous reports. Therefore, it is inevitable that different ADC thresholds were calculated. That was the reason why we also focused on the time to reperfusion from imaging to construct the ML model. Some studies attempted to apply ML to AIS management. It was reported that ML can perform the detection of occlusion sites in CT-A. ML did not contribute to improving the outcome. Meanwhile, our study is a novel study to mention the limited time for MT. We proved the new potential of ML in the management of AIS [35,36,37].

In this study, the pixel-number-based demonstration of ischemic volume did not prove to be a significant feature. This could be attributed to our patient selection criteria, which included only those with TICI 3. Previous research has suggested that even elderly patients with a large ischemic volume may benefit from achieving complete reperfusion [38]. Therefore, there was a possibility that ischemic volume in pre-MT did not reflect outcomes after MT, especially post-mRS after three months. In addition, cases of reversible changes depending on the ADC value have also been observed. The trend may be confirmed by measuring ischemic volume before and after MT. However, since the purpose of this study was also to predict outcomes before MT, postoperative ADC measurements were not performed in this study.

In constructing the ML models, we excluded features from physical findings such as the National Institutes of Health Stroke Scale and preoperative mRS. Because these features clearly significantly affected postoperative outcomes; however, these effects were qualitative, and interobserver errors cannot be ignored. We believe that objectivity can be ensured using features obtained from ADC.

This study had several limitations. First, we used only 75 cases from a single institution. Additionally, we performed data augmentation such as left-right reversal. It would be desirable to construct a model using more real data. Similar investigations should attempt to use larger data sets including data from multicenters. Second, we used an automatic ML method with the Pycaret module in Python. Therefore, we did not perform refining the ML model to enhance the accuracy of the prediction. Thirdly, some patients may not be able to receive MRI examinations due to the contradiction of MRI examination such as metallic implants. Based on the results of this study, we need to try to construct a more detailed and accurate model.

## 5. Conclusions

ADC is the most accessible quantitative value in the imaging examination in pre-mechanical thrombectomy in acute ischemic stroke. Combing the detailed ADC analysis and ML allowed us to predict patient outcomes after the operation. Additionally, the simulation study using the prediction score calculated from the ML model offer the time limit for reperfusion to obtain good outcome such as mRS 0–2. At younger ages, the probability of obtaining a good outcome was high and the time limit to MT is long. Finally, the ML model constructed from the detailed ADC analysis can largely contribute to the application of MT.

## Figures and Tables

**Table 1:** The summary of participants.

*n*	75 (M: 33, F:42)			
	Mean ± SD	Max–Min	Median	IQR
Age	79.9 ± 8.8	46–96	80	[76–86.5]
Image to re-perfusion (min)	121.6 ± 43.9	53–272	117	[87–148]
Pass	1.8 ± 1.1	1–6	1	[1–2]
TICI	3 ± 0	3–3	3	[3–3]
Pre mRS	0.8 ± 1.2	0–4	0	[0–1]
Post mRS	3.5 ± 1.5	0–6	4	[2–5]
NIHSS	17.6 ± 6.7	3–35	18	[13.5–23]
ASPECTS	8.1 ± 2.4	2–11	9	[7–10]

**Table 2:** The intraclass correlation coefficients (ICC) between two measures. High correlations were confirmed among all values. This result showed the robustness of original software in the ADC analysis.

	Thresh Hold	Mean	ICC (1, 2)
Mean of ADC value	620 × 10^−6^ mm^2^/s	566.148	0.99854
	600 × 10^−6^ mm^2^/s	550.277	0.99817
	580 × 10^−6^ mm^2^/s	525.785	0.99924
	560 × 10^−6^ mm^2^/s	505.935	0.99842
	540 × 10^−6^ mm^2^/s	487.790	0.99577
	520 × 10^−6^ mm^2^/s	469.877	0.98503
	500 × 10^−6^ mm^2^/s	457.271	0.98959
	480 × 10^−6^ mm^2^/s	440.193	0.99678
SD of ADC value	620 × 10^−6^ mm^2^/s	149.549	0.99496
	600 × 10^−6^ mm^2^/s	149.758	0.99331
	580 × 10^−6^ mm^2^/s	143.564	0.99323
	560 × 10^−6^ mm^2^/s	138.367	0.99242
	540 × 10^−6^ mm^2^/s	140.507	0.99357
	520 × 10^−6^ mm^2^/s	137.920	0.99159
	500 × 10^−6^ mm^2^/s	133.415	0.99553
	480 × 10^−6^ mm^2^/s	130.750	0.95665
Voxel number	620 × 10^−6^ mm^2^/s	7290.500	0.99982
	600 × 10^−6^ mm^2^/s	5206.500	0.99985
	580 × 10^−6^ mm^2^/s	3283.500	0.99984
	560 × 10^−6^ mm^2^/s	2431.500	0.99986
	540 × 10^−6^ mm^2^/s	1991.500	0.99988
	520 × 10^−6^ mm^2^/s	1278.000	0.99989
	500 × 10^−6^ mm^2^/s	1132.000	0.9999
	480 × 10^−6^ mm^2^/s	897.000	0.99991

**Table 3:** The summary of the comparison of models in Pycaret. The top-performance model was the Extra tree classifier and the second performance model was the Random forest classifier.

Model	AUC	Accuracy	Recall	Prec.	F1
**Extra Trees Classifier**	**0.8983**	**0.8267**	**0.9402**	**0.8383**	**0.8844**
**Random Forest Classifier**	**0.8952**	**0.8447**	**0.9054**	**0.8809**	**0.8912**
Light Gradient Boosting Machine	0.8909	0.8330	0.9141	0.8569	0.8836
Gradient Boosting Classifier	0.8857	0.8330	0.9058	0.8638	0.8827
Ada Boost Classifier	0.8056	0.7907	0.8533	0.8556	0.8503
Logistic Regression	0.7808	0.7424	0.7743	0.8602	0.8043
Decision Tree Classifier	0.7720	0.7902	0.8185	0.8744	0.8439
Linear Discriminant Analysis	0.7471	0.7011	0.7062	0.8502	0.7652
Naive Bayes	0.7450	0.6588	0.6210	0.8567	0.7176
K Neighbors Classifier	0.7341	0.6472	0.6040	0.8555	0.7032
Quadratic Discriminant Analysis	0.6158	0.7604	0.9312	0.7742	0.8434
Dummy Classifier	0.5000	0.3053	0.0000	0.0000	0.0000
SVM–Linear Kernel	0.0000	0.6535	0.6732	0.8143	0.7270
Ridge Classifier	0.0000	0.7189	0.7232	0.8632	0.7794

**Table 4:** The summary of the model performance after tuning for validation data.

Tuned Model	AUC	Accuracy	Recall	Prec.	F1	Kappa	MCC
**Extra Trees Classifier**	0.9178 ± 0.0918	0.8451 ± 0.0675	0.9141 ± 0.0542	0.8708 ± 0.0557	0.8912 ± 0.0486	0.6203 ± 0.1638	0.6268 ± 0.1600
**Random Forest Classifier**	0.9146 ± 0.0754	0.8449 ± 0.0806	0.9225 ± 0.0576	0.8678 ± 0.0736	0.8927 ± 0.0559	0.6086 ± 0.2092	0.6202 ± 0.2000
**Blend model**	0.9076 ± 0.1225	0.8500 ± 0.095	0.9235 ± 0.0695	0.8751 ± 0.0854	0.8962 ± 0.0647	0.6232 ± 0.2507	0.6400 ± 0.2328

**Table 5:** The result of the model performance for test data. The extra tree classifier demonstrated the highest area under the curve (0.833) and accuracy (0.933).

Model	AUC	Accuracy	Precision	Recall	F1 Score
**Extra Trees Classifier**	0.833	0.933	1.000	0.667	0.800
**Random Forest Classifier**	0.750	0.800	0.500	0.667	0.571
**Blend model**	0.750	0.800	0.500	0.667	0.571

## Data Availability

Not applicable.

## Supplementary Materials

The following supporting information can be downloaded at: https://www.mdpi.com/article/10.3390/diagnostics13132138/s1, Table S1: Detailed patient information.
