# Supplementary material for: Predicting Mechanical Thrombectomy Outcome and Time Limit through ADC Value Analysis: A Comprehensive Clinical and Simulation Study Using Machine Learning
Source: Diagnostics (Basel). 2023 Jun 21;13(13):2138. doi: 10.3390/diagnostics13132138 (PMC10340725; doi:10.3390/diagnostics13132138)
Supplement: Supplementary file 1 [file diagnostics-13-02138-s001.zip › diagnostics-2435382-supplementary.pdf]

Supplemental file

n	75 (M: 33, F:42)	
Outcome (Good / Poor)	20 / 55	
Occlusion site	ICA	27
	M1	39
	M2	9
	ACA	1
R / L	38 / 39	
tPA (+ / -)	39 / 36	
	**Mean ± SD**	**Min - Max**
Age	79.87±8.83	46 - 96
Time to re-perfusion from imaging	121.64±43.94	53 - 272
Pass	1.79±1.07	1 - 6
Pre mRS	0.84±1.22	0 - 4
Post mRS	3.52±1.48	0 - 6
NIHSS	17.62±6.71	3 - 35
ASPECTS	8.14±2.42	2 - 11
mean_ADC_620	556.79±40.67	422.75 - 613.88
mean_ADC_600	541.98±39.9	412.86 - 607.54
mean_ADC_580	520.54±40.38	401.18 - 585.07
mean_ADC_560	504.55±39.81	392.93 - 572.57
mean_ADC_540	489.4±41.25	386.23 - 597.5
mean_ADC_520	473.36±43.1	378.04 - 666.66
mean_ADC_500	458.24±42.58	370.38 - 635.75
mean_ADC_480	434.48±64.73	0 - 541.09
SD_ADC_620	152.19±31.92	88.44 - 296.38
SD_ADC_600	151.37±33.1	78.9 - 296.46
SD_ADC_580	147.93±36.65	47.62 - 315.07
SD_ADC_560	145.98±38.89	43.66 - 327.57
SD_ADC_540	144.92±44.42	33.28 - 355.6
SD_ADC_520	144.95±44.67	69.88 - 324.97
SD_ADC_500	141.17±49.66	56.95 - 331.9
SD_ADC_480	137.24±50.4	0 - 357.86
voxel_ADC_620	9574.08±9784.28	23 - 47248
voxel_ADC_600	8144.66±9100.13	11 - 44491
voxel_ADC_580	5846.1±7782.9	0 - 39095
voxel_ADC_560	5005.78±7247.52	0 - 36750
voxel_ADC_540	4273.04±6695.75	0 - 34235
voxel_ADC_520	3652.36±6156.59	0 - 31589
voxel_ADC_500	3117.9±5620.75	0 - 28611
voxel_ADC_480	2661.48±5089.54	0 - 25552

ICA: Internal carotid artery
M1: Middle cerebral artery first portion
M2: Middle cerebral artery second portion
ACA: Anterior cerebral artery
NIHSS: National Institutes of Health Stroke Scale
ASPECTS: Alberta Stroke Programme Early CT Score
